# KINDNESS INTERVENTION AND SELF-PERCEPTION OF AGING: AN INTERGENERATIONAL PERSPECTIVE

**DOI:** 10.1093/geroni/igad104.0251

**Published:** 2023-12-21

**Authors:** Dwight Tse, Ewan Cahill

**Affiliations:** University of Strathclyde, Glasgow, Scotland, United Kingdom; Glasgow Caledonian University, Glasgow, Scotland, United Kingdom

## Abstract

Performing acts of kindness can enhance benefactors’ well-being. However, few studies have examined its impact on older benefactors. This study aimed at evaluating this intervention among older adults and exploring “side benefits” beyond well-being. Specifically, given the negative stereotype on older adults as unproductive and incompetent, we hypothesized that acts of kindness could combat against older adults’ internalized negative self-perceptions and enhance their self-concept. We conducted a randomized controlled experiment with 45 participants in the United Kingdom aged 61-80 (73% female). In the experimental condition, for 14 consecutive days, participants recalled up to three acts of kindness they did for others on that day and planned up to three acts of kindness on the next day, as opposed to listing up to three places they were at in the control condition. Surprisingly, our pre-test post-test comparison revealed no significant difference in well-being improvement between the two conditions. Compared to the control group, the experimental group showed an improvement in attitudes toward own aging, especially for their age-related cognitions in the gain (vs. loss) domain. Beneficiaries were mostly family members (34%), followed by community members (25%) and friends/colleagues (21%). For intergenerational acts of kindness, participants reported similar frequencies of younger and older beneficiaries (compared to their age) and provided predominantly emotional (vs. instrumental) support. Taken together, performing acts of kindness may help older adults counteract negative aging stereotypes and see themselves in a more positive lens as productive and competent members of society.

